# Colonic Metastases from Pleomorphic Carcinoma of the Lung Presenting as an Ileocecal Intussusception

**DOI:** 10.5402/2011/137139

**Published:** 2011-07-09

**Authors:** Sadat Rashid, Dhyan Rajan, Robin Jacob, Keith Dahl, Apsara Prasad, Jaspreet Singh, Ghulam Siddiqui, Venkatesh Sasthakonar, Lester Freedman, Wondwoosen Gebre, Umeko Takeshige, Krishnaiyer Subramani, Kaleem Rizvon, Paul Mustacchia

**Affiliations:** ^1^Department of Gastroenterology, Nassau University Medical Center, 2201 Hempstead Turnpike, East Meadow, NY 11554, USA; ^2^Department of Medicine, Nassau University Medical Center, 2201 Hempstead Turnpike, East Meadow, NY 11554, USA; ^3^Department of Surgery, Nassau University Medical Center, 2201 Hempstead Turnpike, East Meadow, NY 11554, USA; ^4^Department of Pathology, Nassau University Medical Center, 2201 Hempstead Turnpike, East Meadow, NY 11554, USA

## Abstract

Pleomorphic carcinoma is a rare aggressive type of lung cancer that uncommonly metastasizes to the bowel, leading to bleeding, perforation, obstruction, and rarely intussusception. Serving as a lead point, metastatic lesions in the bowel may precipitate intussusception and require immediate surgical intervention. We present a rare case of colonic metastases from a primary lung malignancy, causing ileocecal intussusception in a 57-year old male.

## 1. Introduction

Pleomorphic carcinoma of the lung is rare and accounts for less than 1% of all lung malignancies. Metastasis to the bowel from a primary lung malignancy is a rather uncommon finding and often remains clinically silent. Rarely reported, bowel metastasis may lead to intestinal intussusception, which requires prompt surgical intervention. We describe a case of ileocecal intussusception secondary to metastatic pleomorphic carcinoma of the lung.

## 2. Case Report

A 57-year-old African American male presented to the medical emergency room with complaints of progressively worsening dyspnea for 1 month, productive cough for 2 weeks, and hemoptysis for the past 3 days. Review of symptoms was significant for night sweats and an unintentional weight loss of 30 pounds in the last three months. Past medical history included hypertension, dyslipidemia, and 35 pack-years of cigarette smoking. 

Physical examination was significant for marked pallor, a body mass index (BMI) of 17 kg/m^2^, and decreased breath sounds over the left lung base on chest auscultation.

Laboratory studies revealed a hemoglobin of 4.2 g/dL, hematocrit of 13.2%, white blood cell count of 9,100/mm^3^, and a platelet count of 214,000/mm^3^. Fecal occult blood testing was positive, and there was evidence of iron deficiency anemia.

**Figure 1:**
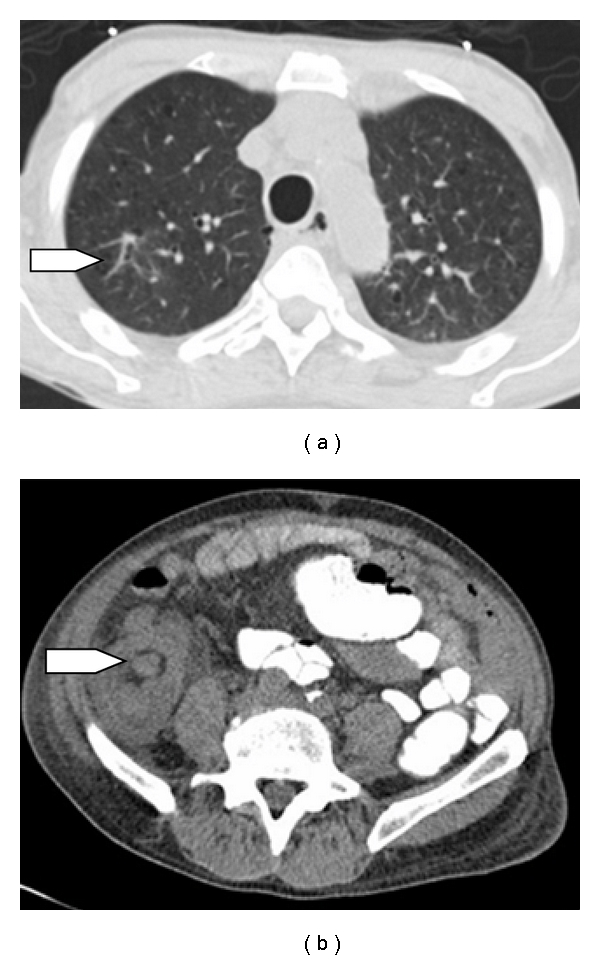
(a) A CT of the thorax revealing a spiculated right upper lobe lung mass. (b) A CT of the abdomen showing ileocecal intussusception.

Chest radiography showed an infiltrate in the left middle and lower lung fields. Subsequent computed tomography (CT) of the chest showed the presence of a 1.9 × 1.6 cm spiculated mass in the right upper lobe suspicious for a neoplasm (Figure 1(a)), with consolidation involving the left lower lobe. 

The patient underwent bronchoscopy that did not reveal any endobronchial lesions, and bronchoalveolar lavage and bronchial brushings were negative for any malignant cells.

Subsequent CT-guided biopsy of the lung mass was positive for non-small cell lung carcinoma (NSCLC).

**Figure 2:**
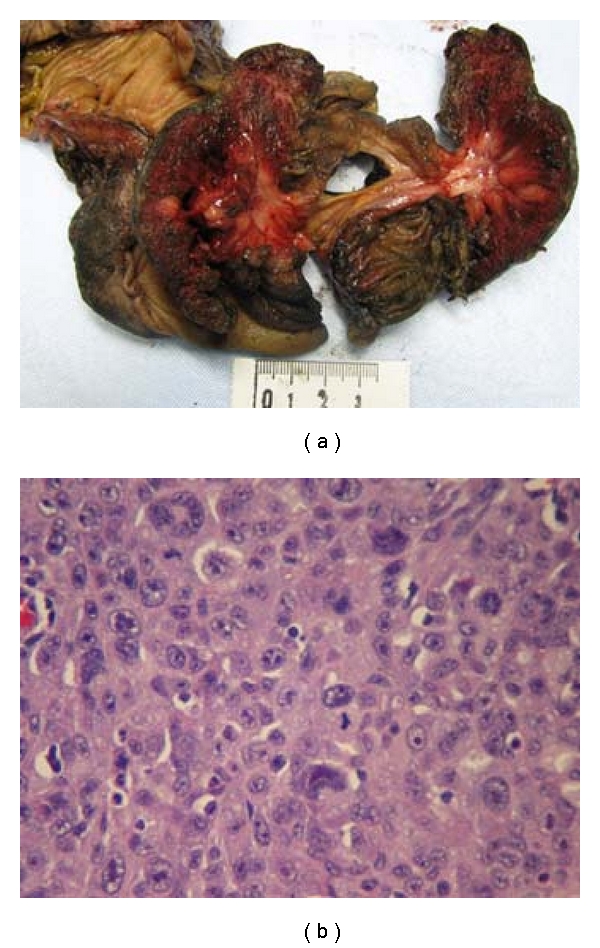
(a) Gross pathology of the surgically resected bowel after right hemicolectomy and ileocolic anastomosis. (b) Histological examination of the cecal tumor, revealing metastatic invasion from pleomorphic carcinoma of lung origin.

During the hospital stay, the patient complained of severe abdominal pain, abdominal distension, and nausea. A CT scan of the abdomen revealed cecal colitis with ileocecal intussusception (Figure 1(b)). In view of his clinical symptoms and CT findings, he underwent an emergent laparotomy. During the surgery a cecal mass with an intussusception involving the terminal ileum and the cecum was identified. A right hemicolectomy with ileocolic anastomosis was performed, and the resected specimen (Figure 2(a)) was sent for histological examination. Surgical pathology revealed metastatic pleomorphic carcinoma of lung origin with giant cell and spindle cell features (Figure 2(b)). Immunohistochemistry results supported the diagnosis of metastatic disease from a primary lung malignancy (Table 1).

Further hospital course was complicated by sepsis, massive hemoptysis, and multiple organ failure which led to the patient's death on day 89 of admission. 

## 3. Discussion

Pleomorphic carcinoma (PC) is categorized by the World Health Organization (WHO) histologic classification of lung tumors as a subtype of heterogeneous group of non-small cell lung carcinomas (NSCLC) that contain a sarcoma or sarcoma-like component under the designation “carcinomas with pleomorphic, sarcomatoid, or sarcomatous elements” (CPSS) [1–3].

Histopathologically, CPSS is a poorly differentiated tumor, with a dual component of sarcomatoid (spindle and/or giant cells), and carcinoma (epithelial cells) elements. Tumors with at least 10 percent of spindle and/or giant cells are considered to be pleomorphic carcinomas. It is a rare lung malignancy that constitutes 0.3 to 1 percent of all malignant lung tumors. This cancer usually occurs in male smokers, with the median age of presentation being 59 years. The tumor usually presents as a peripheral mass predominantly seen in upper lobe of the lung. The prognosis of pleomorphic carcinoma is poor, with a 6-month survival of only 27 percent [4].

Given the lower incidence of pleomorphic carcinoma compared to other tumors of the lung, these may present a diagnostic challenge for pathologists. Nearly half of the cases of pleomorphic carcinoma are diagnosed at an advanced stage, with one-fifth of them already having local chest wall invasion [5].

**Figure 3:**
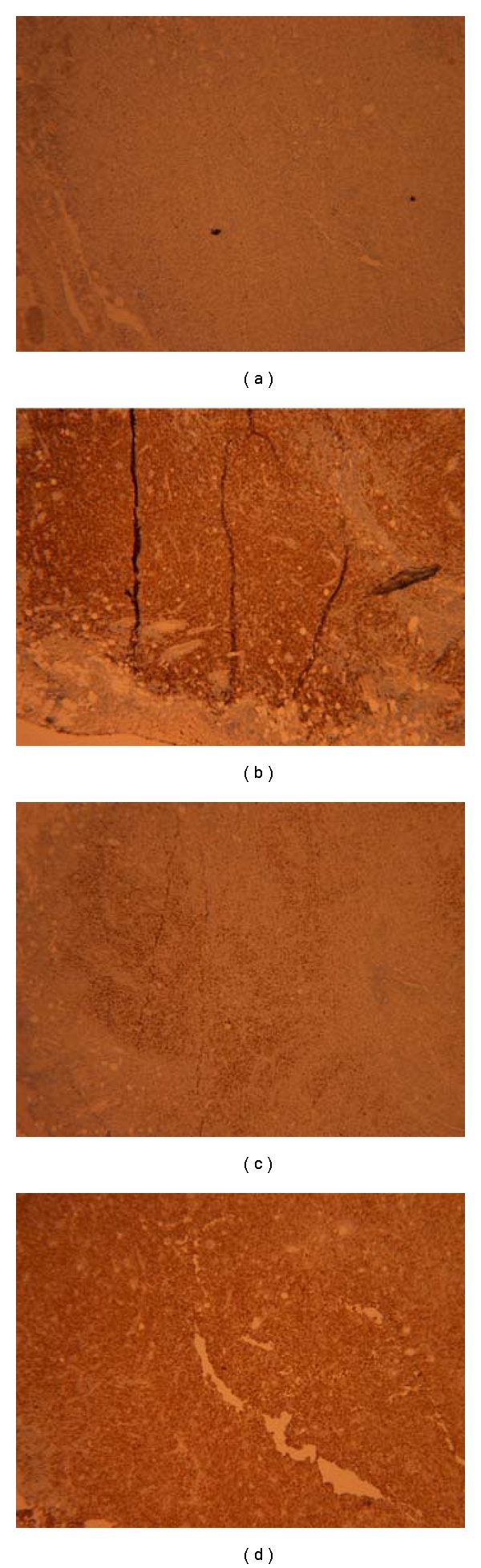
Immunohistochemical staining of the tumor: (a) CK20 negative, (b) CK7 positive, (c) TTF-1 positive, (d) pancytokeratin (AE1/AE3) positive.

The metastasis from pleomorphic carcinoma of the lung to other organs including lymph nodes, brain, bone, liver, adrenals, and contralateral lung have been reported, but metastasis to the gastrointestinal tract remains uncommon [5]. Small bowel metastasis is more common than stomach or colonic metastasis. Most cases of gastrointestinal metastasis have been reported on autopsy studies, as they rarely cause clinical symptoms. Symptomatic bowel metastases can cause bowel obstruction, perforation, gastrointestinal fistula, bleeding, and rarely bowel intussusception. Patients with intestinal metastasis and its complications have a higher mortality and poorer prognosis, often having less than 16 weeks of survival from the time of diagnosis [5]. Immunohistochemistry plays an important role in differentiating metastatic from primary colonic malignancy. Colorectal adenocarcinoma is typically CDX-2 positive, CK20 positive, and CK7 negative. Most primary lung malignancies are TTF-1-positive, CK7 positive and CK20 negative (Figure 3) [6].

Occurring in approximately 1 in 30,000 hospital admissions, the incidence of intussusception is nearly 20-fold more frequent in the pediatric population than in adults [7]. In children, intussusception is usually idiopathic or caused by a viral illness, however adult intussusception is almost always secondary to an organic lesion serving as a lead point [8]. Lesions serving as a leading point may be malignant such as primary adenocarcinoma or lymphomas or may be benign lesions such as lipomas, hamartomas, and postoperative adhesions [9]. Metastatic lesions may also serve as a lead point causing bowel intussusception, however very rarely from a primary lung malignancy. Presenting often with persistent or intermittent abdominal pain, adult intussusception almost always requires surgical intervention which includes surgical exploration and bowel resection.

## 4. Conclusion

Pleomorphic carcinoma is a rare type of lung cancer that infrequently metastasizes to the bowel. Serving as a lead point for possible intussusception, metastatic lesions to the bowel may warrant prompt diagnosis and surgical intervention. Although infrequently reported, one should be aware of the possibility of bowel intussusception from metastatic disease, and suspicion should heighten in patients with lung cancer complaining of abdominal pain.

## Figures and Tables

**Table 1:** Immunohistochemical analysis of the cecal tumor.

Antigen	Result
Pancytokeratin (AE1/AE3)	Tumor 4+
Cytokeratin 7 (CK7)	Tumor 4+
Cytokeratin 20 (CK20)	Tumor negative
CDX2	Tumor negative
Thyroid transcription factor-1 (TTF-1)	Tumor 4+
Vimentin	Tumor positive
Melanocyte antigen (Melan-A)	Tumor negative
Human melanoma black (HMB-45)	Tumor negative
S100	Tumor negative
Pan-melanoma	Tumor negative
Cluster of differentiation 117 (CD117) (c-Kit)	Tumor negative
Desmin	Tumor negative, myofibroblasts positive
Smooth muscle actin (SMA)	Negative
Cluster of differentiation 31 (CD31)	Tumor negative
Cluster of differentiation 34 (CD34)	Tumor negative
Cluster of differentiation 45 (CD45), leukocyte common antigen (LCA)	Tumor negative, entrapped reactive lymphocytes positive
